# Recent advances in gene function prediction using context-specific coexpression networks in plants

**DOI:** 10.12688/f1000research.17207.1

**Published:** 2019-02-05

**Authors:** Chirag Gupta, Andy Pereira

**Affiliations:** 1 Crop, Soil and Environmental Sciences, University of Arkansas, Fayetteville, AR, USA

**Keywords:** context specific, coexpression networks, gene function prediction, network analysis, modules, clusters

## Abstract

Predicting gene functions from genome sequence alone has been difficult, and the functions of a large fraction of plant genes remain unknown. However, leveraging the vast amount of currently available gene expression data has the potential to facilitate our understanding of plant gene functions, especially in determining complex traits. Gene coexpression networks—created by integrating multiple expression datasets—connect genes with similar patterns of expression across multiple conditions. Dense gene communities in such networks, commonly referred to as modules, often indicate that the member genes are functionally related. As such, these modules serve as tools for generating new testable hypotheses, including the prediction of gene function and importance. Recently, we have seen a paradigm shift from the traditional “global” to more defined, context-specific coexpression networks. Such coexpression networks imply genetic correlations in specific biological contexts such as during development or in response to a stress. In this short review, we highlight a few recent studies that attempt to fill the large gaps in our knowledge about cellular functions of plant genes using context-specific coexpression networks.

The most important utility of gene coexpression networks (GCNs) is in expanding the current state of functional annotations of plant genes. The time-honored technique of genetic screens and loss-of-function mutant analyses for gene function characterization has obvious limitations ^1^ and has mapped the functions of only about 24% of all genes in the model plant *Arabidopsis thaliana* (The Arabidopsis Information Resource (TAIR) portal: https://bit.ly/2Ak85Zu). The most alarming fact is that less than 1% of all known genes in important crops like maize and rice have experimentally identified functions ^2, 3^. The less precise orthology-based function assignments are still the default method for newly sequenced plant genomes ^4^. However, despite having a great degree of similarity in sequence or protein domains, genes can evolve for divergent functions ^5^, especially those involved in specialized metabolism (SM) ^6^, leaving younger plant genes less annotated ^7^. Nevertheless, homology-based function annotations are coupled with other experimentally derived manual annotations ^8^ and made available as Gene Ontologies (GOs) for several plant species ^9^. The plant GO and other function annotation catalogs ^10, 11^ provide excellent leads, or *a priori* evidence, to enhance the development of GCNs that offer a scalable and dynamic framework for prediction of gene functions *in silico*.

GCNs are constructed by connecting pairs of genes if they have high (statistically determined) correlation in their expression profiles across a large set of samples ( Figure 1 legend). Coexpressed gene pairs often represent functional coupling, such as through coordinate regulation of specific pathways. Identifying and connecting gene pairs at the genome-wide level are ultimately what reconstruct a network, which is a graphical and mathematical abstraction of functional associations among genes in the cellular states being examined. Much like in community analysis of human social networks ^12^, extraction of densely connected gene communities becomes the next step in gene network analysis. The general idea is that because the genes in each community have a higher degree of coexpression, they are more likely to be under the same regulatory program that dictates their expression and therefore might also be functionally related ^13^. These communities are broadly referred to as “clusters” or “modules” of coexpressed genes.

**Figure 1.**
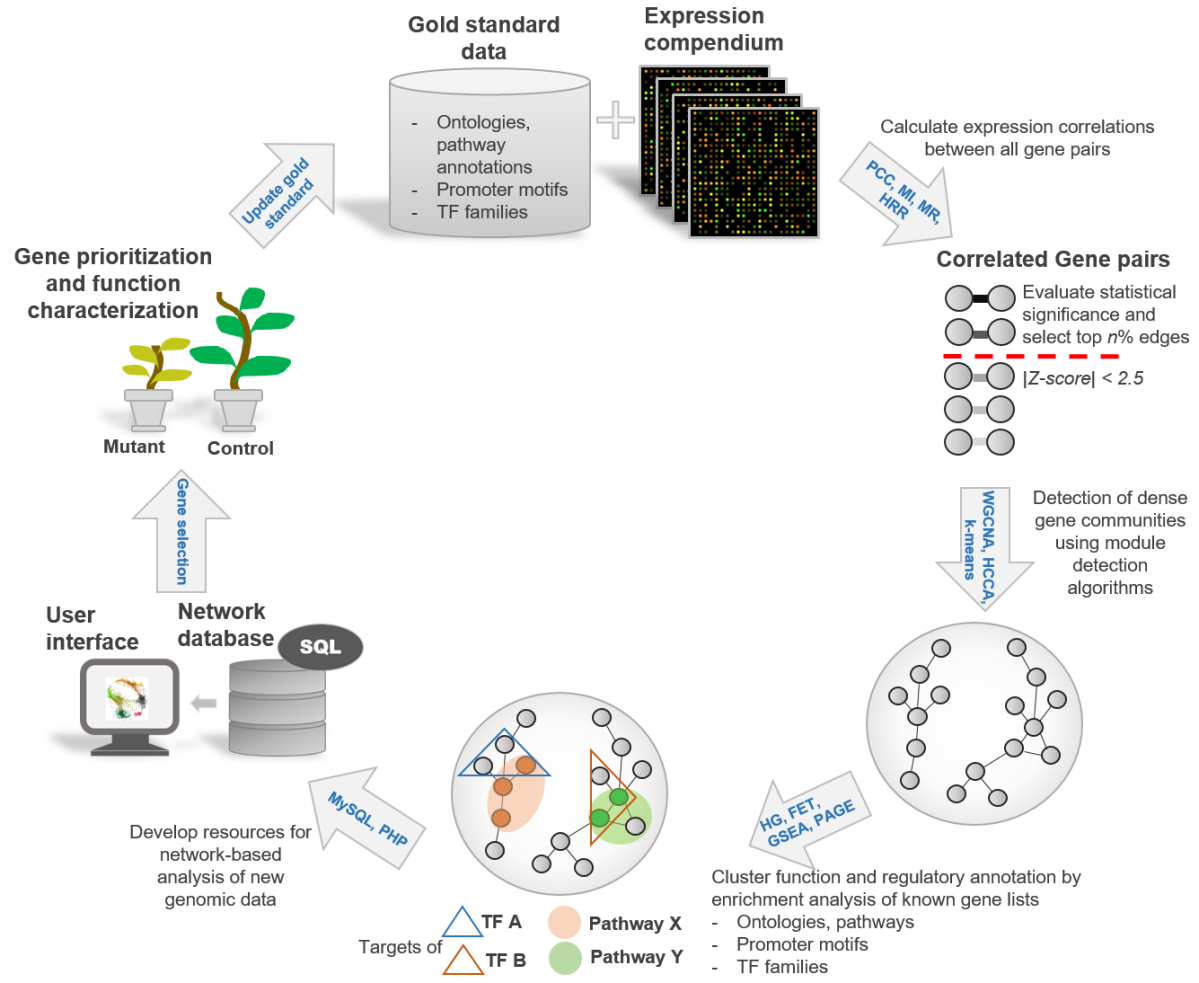
Coexpression network analysis workflow. A gene coexpression network is constructed by integrating gene expression profiles from a large compendium of datasets. The datasets can be sampled from public repositories like the Gene Expression Omnibus ^42^ and quite often are chosen in a manner that represents a unifying biological context (for example, response to abiotic stress or specific tissues/organs of the plant). Correlations in expression profiles of all gene pairs across all samples then are calculated by using a similarity measure such as PCC (Pearson’s correlation coefficient), MI (mutual information), MR (mutual rank) ^30^, or HRR (highest reciprocal rank) ^43^. Statistically significant gene pairs then are linked to each other, and the resulting network is clustered by using a module detection algorithm such as WGCNA (weighted gene coexpression network analysis) in R, HCCA (heuristic cluster chiseling algorithm) ^44^, or the k-means clustering, where k determines the number of clusters. These algorithms identify densely connected network neighborhoods, or modules, that may harbor genes of a common function, pathway, or regulon of a transcription factor (TF) complex. These functional and regulatory attributes of gene modules can be statistically tested by using gene sets from “gold standard” function annotation data. Most often, not all genes within a predicted module have a function annotation, but if the module is significantly enriched with genes known for certain biological process, the functions of other unknown genes can be imputed. Quite often, these data are organized as databases and presented as webtools. (MySQL is a Structured Query Language–based database management system, and PHP is a server-side scripting language.) A community-driven approach is taken to use the data and predict the function(s) of uncharacterized genes. Experimentally validated gene functions then are added to the existing gold standard to further refine the computational predictions in future experiments. The whole process tends to accelerate the process of identifying uncharacterized genes for specific biological processes. FET, Fisher’s exact test; GSEA, gene set enrichment analysis; HG, hypergeometric test; PAGE, parametric analysis of geneset enrichment.

Biological insights from the resulting clustering data can be gained by using well-established computational approaches in gene set analysis (GSA). A wide variety of GSA methods are available that can be used to evaluate whether a predicted module is significantly over-represented with genes already annotated for certain functional categories representing biological processes or pathways ^14– 17^. These predefined functional gene sets for plants can be obtained from annotation catalogs like the GO ^18^, the MapMan bins ^19^, and other pathway databases ^20^. GSA therefore enables propagation of known function information from already annotated genes within each module to genes yet unannotated. However, this technique has several caveats ^21^, and the strength of gene function prediction in this manner depends largely on the quality and expanse of available annotations for the species under consideration. Additionally, a well-designed statistical framework for detecting familiar DNA motifs, or a *de novo* motif search, in the promoters of module genes potentially links transcription factors (TFs) as putative regulators of module expression ^22, 23^.

## Coexpressed gene modules: a treasure trove for plant development and stress biologists

The typical steps involved in construction of GCNs are shown in Figure 1. Some studies report pre-computed global plant gene networks in the form of web applications that provide excellent resources and tools for users to query and visualize subnetworks of interest ^24– 30^. The popular methods, challenges, and caveats associated with construction and use of GCNs are reviewed by others ^31– 33^.

Whereas the construction of a GCN is fairly simple and straightforward, partitioning of a coexpression network into seemingly diverse functional modules is not a trivial task. Several approaches of module detection from gene expression are proposed in the literature and were comprehensively evaluated recently ^34^. Of these methods, weighted GCN analysis (WGCNA) ^35^ has been embraced by plant biologists as the most popular method to identify and analyze gene modules in specific developmental phases of a variety of plant species.

In the context of seed development, Zhan *et al*. ^36^ profiled gene expression in different compartments of maize kernels at the filling stage and identified a module specific to the basal endosperm transfer layer (a tissue layer important for nutrient transport during grain filling). This study also found a link to MRP-1 as a likely regulator of this module by analyzing *cis-*regulatory elements enriched in the promoter of genes in this module ^36^. Similarly, an analysis of modules in developing seeds of two contrasting cultivars of soybean led to the identification of the role of a cytochrome P450 family gene which increases seed size and weight upon overexpression ^37^. In wheat, Wang *et al*. ^38^ identified a module that showed strong association with spike-related traits. The authors of this study validated this module by characterizing three new member genes that showed altered spike complexity when overexpressed in an elite variety of wheat ^38^. An attempt to identify modules of seed development has also been made by using two cultivars of chickpea with contrasting seed size and weight ^39^ and seeds of two different species of cotton ^40^.

To study the development of woodland strawberry ( *Fragaria vesca*) fruits, Shahan *et al*. ^41^ created three separate coexpression networks: one represented early-stage floral tissues, another spanned the development of pre-fertilized flower to fruits, and the third represented fruits at the ripening stages ^41^. These networks were helpful in establishing the role of the ghost tissue in iron transport post-fertilization ^41^. To mine these networks, the authors of this study built an interactive web interface, which is of value to other users in the field ( www.fv.rosaceaefruits.org).

These recent studies have highlighted the efficacy of the WGCNA as a quick and easy method to process new, high-resolution developmental transcriptomes and recover plant gene modules. However, assessing the stability of predicted modules and performance in terms of accuracy is highly desirable for generating new hypotheses ^45, 46^. For example, Shahan *et al*. ^41^ found little overlap between clusters obtained by multiple WGCNA runs each with a different subsampling of genes. Through various numerical experiments, the authors suggest that a consensus clustering scheme is much more robust in terms of predicting true functional relationships between genes ^41^. Also, the WGCNA method implicitly decides on the number of modules to produce from the dataset and, as evident from the aforementioned studies, typically retrieves very few modules (about 10–20 modules per dataset) in a non-exhaustive manner under default settings. It is still unclear whether this is due to the nature of underlying datasets or whether other relevant modules (or subnetworks within the identified WGCNA modules) still exist in these development networks. We believe that a vast majority of plant transcriptomes are still under-utilized in the retrieval of biologically relevant information. Secondary analyses of these datasets can reflect on new knowledge pertaining to the originally assayed biological processes, especially the role of regulatory genes ^47, 48^, which is often overlooked in terms of the potential in typical GCN analysis.

In another type of module identification setting, the goal is to cluster a GCN by using a graph clustering algorithm optimized to detect a large number of dense modules. This type of study aims to partition the GCN into many functionally cohesive gene modules, typically in the range of a few hundred modules per network. This is a desirable property of biological network analysis, and several algorithms exist that allow the user to define module size as desired ^44, 49, 50^. A large number of dense modules would likely separate large metabolic pathways and biological processes into their constituent parts. Intuitively, an important cautionary aspect of such an analysis is controlling the granularity of resulting modules ^51^. Very large modules will fail to answer the fundamental question of why the genes are clustered that way. It becomes difficult to explain the regulatory cohesiveness of large modules, as these will incorporate genes that lie on distant bifurcated branches of large metabolic pathways that show significant levels of correlation in expression ^52^. Conversely, a finer granularity will break the network into too many very small modules (fewer than 10 genes), essentially hampering the statistical process of establishing any functional or regulatory context for these modules, rendering them unusable.

A balance between the number of modules, average module size, and other network topological features such as separation (how functionally isolated each module is from other modules) and density (how well genes within each module form a clique) should be carefully monitored by tuning parameters of the clustering algorithm ^34, 53^. For example, modules in the Rice Environment Coexpression Network (RECoN) ^26^ were identified by optimizing the parameters of a graph-based clustering algorithm that works under the principle that functionally related genes have denser connections within a network ^50^. With controlled minimum cluster size and density threshold to partition the network, 1744 dense abiotic stress–associated rice modules were extracted from RECoN. Most of these modules contain genes sharing related GO terms and thus can be used as gene sets (in addition to gene sets from GO and other function databases) in enrichment analysis of new differential expression profiles. The RECoN webtool (available at https://bit.ly/2BOky7x) also provides a window to mine undiscovered novel modules associated with tolerance to abiotic stresses in rice ^26^.

An example of a graph-based clustering algorithm applied to a developmental context in rice is the RiceAntherNet, which delineated 545 modules related to anther and pollen development ^54^. Of these, 29 modules that contain differentially expressed genes in nine previously known male sterile mutant lines are regarded as important to anther development ^54^. This study also compared the rice anther modules to modules in FlowerNet, a GCN for anther and pollen development in Arabidopsis ^55^, and found a significant amount of conservation of gene coexpression between rice and Arabidopsis during anther development.

GCNs have also been instrumental in the discovery of novel biological phenomena. For example, the conservation of a longevity module between *Medicago truncatula* and Arabidopsis suggests conserved genetic pathways related to defense mechanisms ^56^. Phylogenetic conservation of coexpression modules was examined in greater detail recently for several model plants ^7^. One of the observations of this study was that genes from the same phylostratum tend to be more frequently connected in GCNs ^7^, suggesting new uses of existing GCNs in extrapolating gene functions of lesser-studied plant species. Moreover, it has been shown that coexpression, rather than physical proximity on chromosomes (biosynthetic gene clusters), is a more reliable signal for predicting genes involved in SM ^57^. Understanding the mechanisms of SM is of great interest in the study of medicinal plants ^58^.

## The curious case of transcription factors

Another desirable characteristic of coexpression network modeling is incorporating information about regulatory interactions in the process of module identification. This type of study leads to the discovery of modular gene regulatory networks (GRNs). GRNs are different from GCNs in the sense that GCNs treat TF and non-TF nodes (genes) similarly whereas GRN involves sophisticated reverse-engineering algorithms that operate on TFs differently. The objective of a GRN is to capture direct, causal edges between TF and their putative targets and filter spurious indirect correlations that naturally arise in coexpression data.

Biological interpretations of predicted GRNs depend largely on the type of transcriptional dataset used. A meta-analysis by Marbach *et al*. ^59^ shows that expression data from TF knockouts or overexpression experiments can be very informative in predicting targets but this type of data is useful mostly for capturing downstream regulatory effects ^60, 61^. On the other hand, time-series expression data can capture temporal dynamics of regulatory networks more systematically ^62, 63^. For example, a time-series transcriptome dataset was used to predict GRNs active for lateral root initiation in Arabidopsis, which revealed genetic cascades involved in positive and negative feedback loops as well as target genes of the AUXIN RESPONSE FACTOR 7 ^64^. Steady-state expression data have also been very useful in regulatory network analysis and predicting TF function. For example, using a collection of gene expression datasets from Arabidopsis seeds, subnetworks associated with desiccation tolerance (DT) were predicted, leading to the identification of three novel TFs now confirmed for their roles in mediating seed DT ^65^.

Recently, de Luis Balaguer *et al*. ^66^ developed a dynamic Bayesian network (DBN)-based framework which showed us a broader picture of the spatiotemporal dynamics of stem cell differentiation in the roots of Arabidopsis. Although DBNs are able to unfold cyclic processes and dependencies in time-course expression data, they are inherently limited by the computational complexity which increases with the number of genes ^61^. The algorithm proposed by de Luis Balaguer *et al*. ^66^ seems to circumvent this limitation by applying DBNs to smaller sets of genes within each spatially distinct coexpressed cluster within the roots. This combination of spatial and temporal expression data in GRN inference established a new role of PERIANTHIA (PAN) as a stem cell regulator ^66^.

There are more than a dozen other published algorithms for inference of GRNs from large-scale expression data. Several concepts in GRN inference, available algorithms, and their limitations and applications in plant studies are well summarized by others as a primer to interested researchers ^61, 63, 67, 68^. An earlier meta-analysis of some of the popular approaches suggests integrating predictions from different algorithms to boost the accuracy of the consensus GRN ^59^. This technique was later implemented in Arabidopsis stress datasets to predict an oxidative-stress GRN ^69^ and was further explored in the development of a secondary cell wall biosynthesis network ^70^. The authors of the former study used “coregulated” gene pairs instead of “coexpressed” gene pairs into the k-means clustering framework and recovered 572 stress-related modules ^69^.

Because a large sample size can be obtained for Arabidopsis, it has been a favorite model in many currently reported GRNs. However, rapidly expanding data repositories allow selection of interest-appropriate transcriptional datasets ^71^, encouraging the application of standard GRN inference techniques to relatively lesser-studied crop genomes. For example, Xiong *et al*. ^47^ re-analyzed the maize embryo and endosperm development dataset ^72^ and predicted modules and regulators involved in the transport of nutrients to the developing seed ^47^.

Inference of GRNs can be further enhanced in a direct network framework (non-modular GRNs), where the primary goal is to explore regulatory targets of a few TFs of interest in more detail as focused small-scale subnetworks. For example, a combination of transcriptional and chromatin immunoprecipitation data (ChIP-seq) revealed GRN components involved in coordinate regulation of root hair growth in Arabidopsis ^73^. Furthermore, an integrative analysis of ChIP-seq, mRNA-seq, and miRNA-seq data revealed SEP3 as an upstream regulator of *MIR319a* and *TCP4*, which together form a feed-forward loop to regulate petal development ^74^. In the context of GRNs that manifest during abiotic stresses, Wilkins *et al*. ^75^ employed the concept of ERGINs (environmental gene regulatory influence networks) in rice. They integrated chromatin accessibility data (ATAC-seq) with the current state of knowledge about putative regulatory interactions in rice and used these data points as *priors* to learn a GRN from expression dataset of five tropical Asian rice cultivars grown under abiotic-stress conditions in the field as well as greenhouses. The study identified regulatory interactions between 113 TFs and 4052 target genes of rice ^75^.

## Conclusions

The current state of accumulated plant gene expression data has immense potential for the discovery of components involved in complex traits. The property of modularity in gene networks can be exploited and gene modules treated as fundamental biological units with dynamic expression and regulatory properties. The techniques of module extraction have proven to be very effective in experimental validations while also suggesting a vast scope for improvement in terms of not only statistical methods used but also how gene networks are perceived and evaluated for research. As noticed by Gillis and Pavlidis ^21^, one has to consider that there are several caveats associated with using prior knowledge from GO (and other such annotation catalogs) for function prediction from GCN modules. Predictions can be very biased toward genes and categories that are extremely well annotated and can be driven solely by other computationally predicted annotations rather than empirical evidence.

Transcriptome datasets integrated in a global manner capture broad, constitutive functional relationships that might not vary much with different tissues or organs, developmental phases, or environmental cues like biotic or abiotic stress ^67, 76^. On the other hand, specifying an overarching biological theme in selection of datasets offers intuitive concepts that can be objectively tested. For example, just like individual transcriptomes, GCNs created to study one particular biological process (for example, seed development or response to abiotic stress) can be considered static. Comparison of GCNs constructed from conditionally distinct samples, or differential coexpression analysis, will provide valuable information on how plant systems alter their mechanisms in response to different developmental cues and environmental perturbations ^77, 78^. Moreover, a comparison of sets of modules derived under different contexts should potentially map and distinguish modules that are conserved throughout growth and development from those that are under constant rewiring.

One major question that remains is how to systematically produce a ranked list of genes most relevant to a given trait/process of interest from these complex interconnected gene relationships in networks. Information buried within hundreds of thousands to sometimes millions of predicted functional relationships is not intuitively tractable for researchers interested in selecting a few actionable candidate genes relevant to a biological process of interest. Research toward development of computational tools capable of using gene networks to systematically enrich gene prioritization pipelines ^79^ would be extremely useful for integration with gene lists from genome-wide association study (GWAS) datasets in a systems genetics approach to probe complex agricultural traits ^80, 81^.

It is important to recognize that gene expression data by itself could have limited potential in deciphering cellular organization, regulated at various levels. However, we are optimistic about the future, as integrating signals from heterogeneous molecular datasets will enable training of smart algorithms to identify genomic patterns of components already known to be associated with a phenotype of interest. The trained models then can be used as predictive tools to discover new genes associated with the phenotype ^82^ and study crop genetics as outlined in recent reviews ^83, 84^.
